# Association of Medicaid vs Marketplace Eligibility on Maternal Coverage and Access With Prenatal and Postpartum Care

**DOI:** 10.1001/jamanetworkopen.2021.37383

**Published:** 2021-12-06

**Authors:** Erica L. Eliason, Jamie R. Daw, Heidi L. Allen

**Affiliations:** 1 Columbia University School of Social Work, New York, New York; 2 Columbia University Mailman School of Public Health, New York, New York

## Abstract

**Importance:**

Policy makers are considering insurance expansions to improve maternal health. The tradeoffs between expanding Medicaid or subsidized private insurance for maternal coverage and care are unknown.

**Objective:**

To compare maternal coverage and care by Medicaid vs marketplace eligibility.

**Design, Setting, and Participants:**

A retrospective cohort study using a difference-in-difference research design was conducted from March 14, 2020, to April 22, 2021. Maternal coverage and care use were compared among women with family incomes 100% to 138% of the federal poverty level (FPL) residing in 10 Medicaid expansion sites (exposure group) who gained Medicaid eligibility under the Affordable Care Act and in 5 nonexpansion sites (comparison group) who gained marketplace eligibility before (2011-2013) and after (2015-2018) insurance expansion implementation. Participants included women aged 18 years or older from the 2011-2018 Pregnancy Risk Assessment Monitoring System survey.

**Exposures:**

Eligibility for Medicaid or marketplace coverage under the Affordable Care Act.

**Main Outcomes and Measures:**

Outcomes included coverage in the preconception and postpartum periods, early and adequate prenatal care, and postpartum checkups and effective contraceptive use.

**Results:**

The study population included 11 432 women age 18 years and older (32% age 18-24 years, 33% age 25-29 years, 35% age ≥30 years) with incomes 100% to 138% FPL: 7586 in a Medicaid state (exposure group) and 3846 in a nonexpansion marketplace state (comparison group). Women in marketplace states were younger, had higher educational level and marriage rates, and had less racial and ethnic diversity. Medicaid relative to marketplace eligibility was associated with increased Medicaid coverage (20.3 percentage points; 95% CI, 12.8 to 30.0 percentage points), decreased private insurance coverage (−10.8 percentage points; 95% CI, −13.3 to −7.5 percentage points), and decreased uninsurance (−8.7 percentage points; 95% CI, −20.1 to −0.1 percentage points) in the preconception period, increased postpartum Medicaid (17.4 percentage points; 95% CI, 1.7 to 34.3 percentage points) and increased adequate prenatal care (4.4 percentage points; 95% CI, 0.1 to 11.0 percentage points) in difference-in-difference models. No evidence of significant differences in early prenatal care, postpartum check-ups, or postpartum contraception was identified.

**Conclusions and Relevance:**

In this cohort study, eligibility for Medicaid was associated with increased Medicaid, lower preconception uninsurance, and increased adequate prenatal care use. The lower rates of preconception uninsurance among Medicaid-eligible women suggest that women with low incomes were facing barriers to marketplace enrollment, underscoring the potential importance of reducing financial barriers for the population with low incomes.

## Key Points

**Question:**

How does preconception and postpartum coverage and care use compare among women with low incomes gaining eligibility for Medicaid vs marketplace coverage?

**Findings:**

In this cohort study of 11 432 women with low incomes, Medicaid eligibility relative to marketplace eligibility was associated with significantly increased Medicaid coverage (20.3 percentage points), decreased private insurance coverage (−10.8 percentage points), and decreased uninsurance (−8.7 percentage points) in the preconception period. It was also associated with increased postpartum Medicaid (17.4 percentage points) and increased adequate prenatal care (4.4 percentage points) but not with significant changes in early prenatal care, postpartum checkups, or postpartum contraception.

**Meaning:**

The finding of lower rates of preconception uninsurance among Medicaid-eligible women in this study suggests that women with low incomes were facing barriers to marketplace enrollment.

## Teaser

This cohort study examines changes in Medicaid vs marketplace prenatal and postpartum coverage of women with low incomes in states with vs without Medicaid expansion under the Affordable Care Act.

## Introduction

Insurance coverage is necessary for ensuring access to health care before and after childbirth, yet women in the US have high rates of discontinuous insurance in the perinatal period.^1^ Insurance expansions are a potential policy lever for improving maternal health in the US.^2,3^ The Affordable Care Act (ACA) created a unique opportunity to evaluate the outcomes associated with gaining eligibility for private coverage vs Medicaid and maternal health. Adults with family incomes between 100% to 138% of the federal poverty level (FPL) either gained Medicaid eligibility if they lived in a state that adopted Medicaid expansion or gained eligibility for subsidized private marketplace coverage if they lived in a nonexpansion state. Studies that exploited this natural experiment found that, compared with marketplace eligibility, Medicaid eligibility was associated with lower uninsurance and out-of-pocket spending but more difficulty finding and accessing care and longer wait times.^4,5^ Studies examining ACA Medicaid expansions and the dependent coverage provision found reduced preconception and postpartum uninsurance and small or no improvements in prenatal care; however, these studies have not explicitly compared public vs private insurance eligibility.^6,7,8,9,10^ Thus, the objective of this study was to compare the implications of gaining eligibility for Medicaid vs gaining eligibility for subsidized private coverage through the marketplace on maternal coverage and access to prenatal and postpartum care among women with low incomes.

## Methods

### Data and Sample

We used data from the 2011-2018 Pregnancy Risk Assessment Monitoring System (PRAMS), a multistate survey conducted by state and city departments of health in collaboration with the Centers for Disease Control and Prevention. PRAMS conducts surveys of respondents with a live birth about their experiences before, during, and after pregnancy.^11^ Consistent with PRAMS, we use the term *women* for this sample; however, no gender-identity questions are asked. We limited the sample to 14 states and New York City with consistent reporting in PRAMS throughout the study period. Of these jurisdictions, 10 adopted Medicaid expansion by 2018 (Colorado, Delaware, Massachusetts, New Jersey, New Mexico, New York City, Pennsylvania, Vermont, Washington, and West Virginia) and 5 did not (Maine, Missouri, Utah, Wisconsin, and Wyoming) (eTable 1 in the Supplement). To compare Medicaid eligibility (if they resided in a Medicaid expansion state) or marketplace eligibility (if they resided in a nonexpansion state), we limited our analysis to women aged 18 years or older whose family incomes were 100% to 138% of the FPL. We calculated the FPL using the midpoint of the self-reported income category and the number of dependents, following methods used in prior PRAMS analyses.^7^ This study was considered to be not human subjects research and the need for approval was waived by the Columbia University Institutional Review Board. This study followed the Strengthening the Reporting of Observational Studies in Epidemiology (STROBE) reporting guideline.

### Outcomes

Our primary outcomes were insurance coverage and prenatal and postpartum care use. We measured insurance at 2 periods: preconception, defined as 1 month before pregnancy, and post partum, defined as insurance status at the time of the PRAMS survey, which ranged from 0 to 15 months after birth (mean, 4 months). Following previous classifications, we categorized insurance status into mutually exclusive categories: Medicaid, private coverage, or uninsurance.^1,12^ The Medicaid category included Medicaid alone or in combination with other coverage. The private insurance category included insurance purchased individually, marketplace insurance, employer-sponsored insurance, insurance through a parent, TRICARE or military insurance, or other insurance. The uninsured category comprised individuals with no insurance or care through the Indian Health Service, consistent with previous insurance groupings of PRAMS coverage categories.^13^

We examined early prenatal care (ie, first visit in the first trimester), adequate prenatal care (Kessner index), receipt of a postpartum checkup, and use of effective postpartum contraception.^14,15^ The Kessner index is a composite measure of early prenatal care initiation and total number of visits. Effective postpartum contraception included sterilization, intrauterine devices, implants, injectables, oral contraceptive, transdermal patch, or vaginal ring. Postpartum insurance measures were available beginning in 2012; all other outcomes were available from the start of the study period (2011). The study was conducted from March 14, 2020, to April 22, 2021.

### Statistical Analysis

We used a difference-in-difference research design to compare the change in outcomes from the prepolicy to the postpolicy period for women with incomes 100% to 138% of the FPL in Medicaid expansion vs marketplace states, consistent with previous research comparing the association of gaining eligibility for Medicaid vs subsidized private marketplace coverage.^4,5^ We excluded the first year after Medicaid expansion as a transition year (2014 in all states except Pennsylvania, which implemented Medicaid expansion in January 2015). The sensitivity of our analyses to omitting the transition year was explored by including the transition period (eTable 5 in the Supplement). We ran linear probability models including state and year fixed-effects and adjusting for mother’s age, marital status, race and ethnicity, educational attainment, and state-level rates of unemployment for women from the Bureau of Labor Statistics. We included race and ethnicity variables because of the racial and ethnic disparities in maternal health outcomes in the US, and because of previous research indicating differential effects of health insurance expansions on maternal outcomes.^2^ Race and ethnicity categories were self-reported and available from the birth certificate files. These categories were American Indian/Alaskan Native, Asian/Pacific Islander, Black, Hispanic, White, and Other/mixed (mixed race was combined with Other non-White for the Other category). The association of gaining Medicaid relative to marketplace eligibility was measured using a binary variable indicating whether a given state-year was postpolicy and whether the state was a Medicaid expansion state (eTable 2 in the Supplement). We created missing categories for all control variables with missing data. We used wild bootstrap SEs clustered by state to account for the small number of clusters.^16^ We used PRAMS analytic weights provided by the Centers for Disease Control and Prevention to account for sampling design, nonresponse, and noncoverage in the survey. We used 2-sided statistical tests and a significance level of *P* < .05.

The difference-in-difference approach assumes that the counterfactual temporal trends in the outcomes would not have differed by state expansion status if not for the ACA implementation. Using prepolicy data (2011-2013), we tested the plausibility of this parallel trends assumption by estimating each outcome as a function of a linear time trend interacted with Medicaid expansion status along with control variables (eTable 2 in the Supplement). The unadjusted trends in private and Medicaid coverage for the preconception and postpartum periods are presented by Medicaid or marketplace eligibility in eFigure 1 in the Supplement. We conducted placebo tests for the prepolicy period, analyzing the difference in outcomes in Medicaid vs marketplace states for each year compared with the last year of the prepolicy period (2013), controlling for covariates. We present *F* statistics for joint tests of the null hypothesis that all prepolicy interaction terms are equal to 0 to further investigate evidence of violation of the parallel trends assumption (eTable 3 in the Supplement). Event studies are displayed in eFigure 2 in the Supplement to explore trends in outcomes between expansion and marketplace states relative to the last year of the prepolicy period.

In sensitivity analyses, we included difference-in-difference models that excluded women aged 18 to 26 years, as this group may have gained insurance under the dependent coverage provision and thus would not have been as affected by the 2014 insurance expansions (eTable 6 in the Supplement). We additionally included difference-in-difference models that omit states with pre-ACA Medicaid eligibility up to at least 100% of the FPL (New York, Massachusetts, Vermont, and Delaware)^17^ and used a complete case analysis as an alternative approach to addressing missing data (eTable 7 and eTable 8 in the Supplement). In addition to preconception and postpartum insurance measures, PRAMS includes coverage at delivery from the birth certificate files. This measure is included in eTable 10 in the Supplement as an additional outcome.

## Results

We identified a total of 11 432 women age 18 years and older (32% age 18-24 years, 33% age 25-29 years, 35% age ≥30 years) with incomes 100% to 138% FPL: 7586 resided in a Medicaid state and 3846 resided in a marketplace state. Table 1 presents demographic characteristics for the prepolicy period by state Medicaid expansion status. Marketplace states had higher rates of women who were aged 18 to 24 years (40.4% vs 33.7%), married (58.5% vs 52.0%), had educational attainment beyond high school (55.9% vs 49.0%), and a race and ethnicity of non-Hispanic White (73.2% vs 43.8%) compared with Medicaid expansion states. Overall, in the prepolicy vs postpolicy periods, there were 109 (1.3%) vs 35 (0.8%) American Indian/Alaskan Native, 223 (7.2%) vs 50 (2.8%) Asian/Pacific Islander, 486 (15.2%) vs 244 (7.0%) Black, 708 (27.9%) vs 265 (12.7%) Hispanic, 1440 (43.8%) vs 973 (73.2%) White, and Other/mixed (105 (2.7%) vs 66 (2.9%) women.

**Table 1.** Demographic Characteristics of Women at 100% to 138% of the Federal Poverty Level by Eligibility for Medicaid or Marketplace Insurance^a^

Demographic characteristics	Medicaid expansion states (Medicaid eligible)	Medicaid nonexpansion states (marketplace eligible)	*P* value
No.	3389	1645	
Age, y			
18-24	1194 (33.7)	668 (40.4)	<.001
25-30	976 (31.4)	507 (32.8)	
30-34	743 (21.9)	323 (19.3)	
35-39	370 (10.2)	109 (6.0)	
40 or older	106 (2.7)	37 (1.4)	
Missing	0	1 (0.2)	
Marital status			
Married	1719 (52.0)	940 (58.5)	<.001
Not married	1664 (47.9)	704 (41.5)	
Missing	6 (0.1)	1 (0.0)	
Race/ethnicity			
American Indian/Alaskan Native	109 (1.3)	35 (0.8)	<.001
Asian/Pacific Islander	223 (7.2)	50 (2.8)	
Black	486 (15.2)	244 (7.0)	
Hispanic	708 (27.9)	265 (12.7)	
White	1440 (43.8)	973 (73.2)	
Other/mixed^b^	105 (2.7)	66 (2.9)	
Missing	318 (2.6)	12 (0.9)	
Educational level			
High school or less	1684 (50.5)	788 (42.9)	<.001
More than high school	1681 (49.0)	813 (55.9)	
Missing	24 (0.5)	44 (1.2)	

^a^ Unweighted numbers and weighted percent estimates are presented, with data weighted using Pregnancy Risk Assessment Monitoring System sample weights. Results of χ^2^ tests are presented to assess differences in characteristics by eligibility for Medicaid or marketplace insurance.

^b^ Race and ethnicity are self-reported. Other/mixed includes other non-White and mixed race.

**Figure 1.**
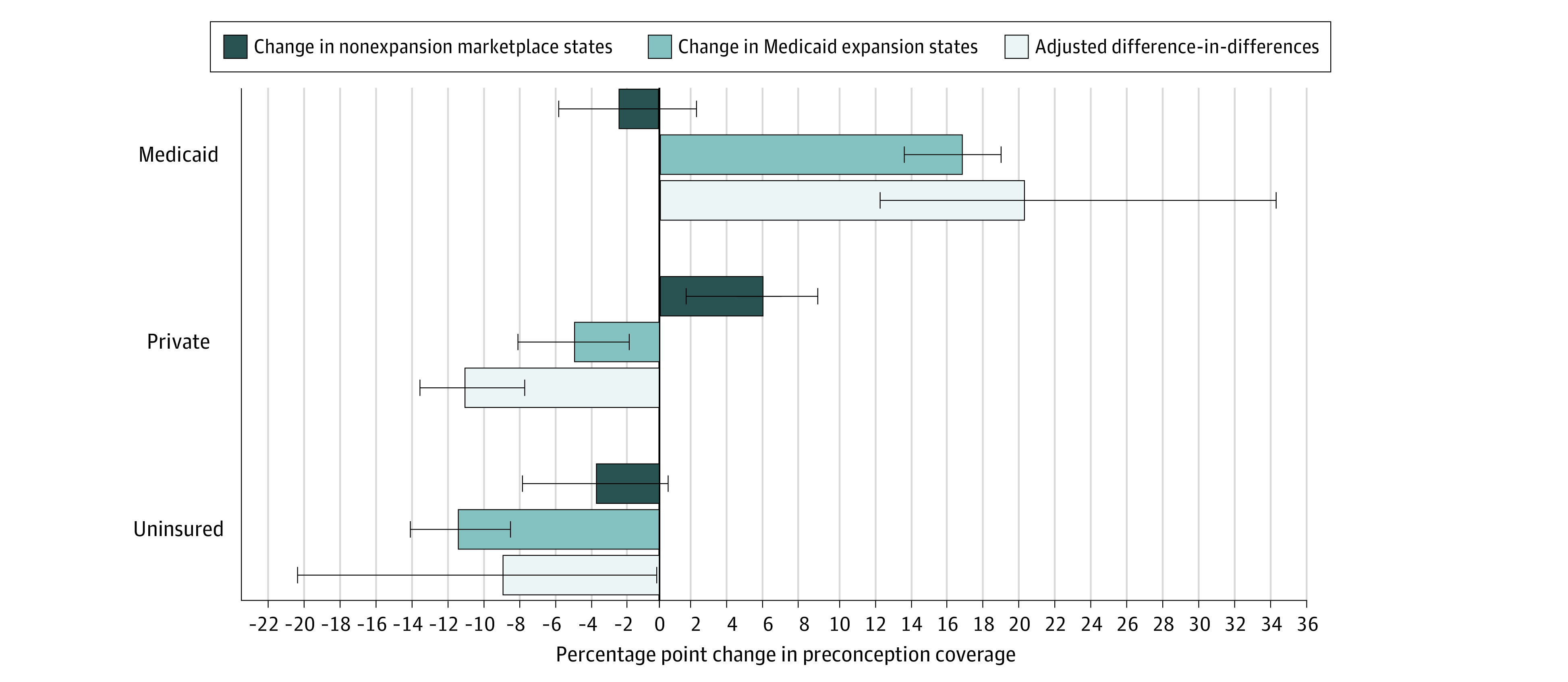
Changes in Preconception Coverage Among Low-Income Women at 100% to 138% of the Federal Poverty Level, by Medicaid or Marketplace State Eligibility Analysis of Pregnancy Risk Assessment Monitoring System (PRAMS) data from 14 states and New York City (N = 11 432). Data were weighted using PRAMS sample weights. Error bars represent 95% CIs.

Figure 1 displays the unadjusted changes in preconception coverage in Medicaid and marketplace states and the adjusted difference-in-differences estimate in Medicaid relative to marketplace states. In adjusted models, Medicaid relative to marketplace eligibility increased preconception Medicaid (20.3 percentage points; 95% CI, 12.8 to 30.0) and decreased preconception private coverage (−10.8 percentage points; 95% CI, −13.3 to −7.5), with a net decrease of preconception uninsurance (−8.7 percentage points; 95% CI, −20.1 to −0.1 percentage points) (Table 2). At delivery, we found higher Medicaid coverage and lower private coverage with no net change in uninsurance relative to marketplace eligibility (eTable 10 in the Supplement).

**Table 2.** Estimates of the Association of Medicaid vs Marketplace Eligibility With Maternal Coverage and Access to Prenatal and Postpartum Care^a^

Dependent variable	No. (%)				Difference-in-difference estimates of Medicaid vs marketplace eligibility^b^			
	Medicaid nonexpansion states (marketplace eligible)		Medicaid expansion states (Medicaid eligible)					
	Prepolicy, 2011-2013 (n = 1645)	Postpolicy, 2015-2018 (n = 2201)	Prepolicy, 2011-2013 (n = 3389)	Postpolicy, 2015-2018 (n = 4197)	Unadjusted, percentage points (95% CI)	*P* value	Adjusted, percentage points (95% CI)	*P* value
Preconception coverage								
Medicaid	502 (27.1)	650 (25.4)	1099 (33.1)	2273 (49.9)	20.1 (12.3 to 30.3)	.003	20.3 (12.8 to 30.0)	.002
Private	609 (40.5)	943 (46.3)	1018 (32.2)	1123 (27.5)	−10.2 (−13.8 to −6.0)	.001	−10.8 (−13.3 to −7.5)	.001
None	511 (30.8)	593 (27.3)	1019 (29.7)	638 (18.6)	−8.9 (−20.7 to −0.1)	.05	−8.7 (−20.1 to −0.1)	.05
Postpartum coverage								
Medicaid	452 (41.7)	931 (39.5)	1077 (46.3)	2777 (61.6)	17.5 (2.8 to 37.3)	.01	17.4 (1.7 to 34.3)	.02
Private	326 (33.1)	753 (37.3)	505 (25.6)	826 (20.9)	−8.2 (−17.2 to 4.5)	.25	−8.6 (−16.8 to 3.7)	.24
None	268 (24.3)	490 (22.0)	476 (22.3)	438 (13.2)	−7.0 (−18.5 to 5.1)	.22	−6.5 (−17.7 to 4.2)	.20
Prenatal and postpartum care use								
Early prenatal care	1337 (80.8)	1826 (83.4)	2665 (80.0)	3457 (83.7)	1.3 (−3.8 to 5.9)	.54	1.0 (−4.6 to 8.4)	.70
Adequate prenatal care	1127 (75.4)	1477 (74.2)	2169 (65.6)	2771 (70.0)	5.4 (0.1 to 12.5)	.05	4.4 (0.1 to 11.0)	.05
Postpartum checkup	1307 (90.5)	1903 (86.9)	2460 (89.6)	3630 (87.6)	0.6 (−3.3 to 4.2)	.75	0.3 (−3.1 to 3.9)	.83
Effective postpartum contraception	699 (43.5)	1245 (54.2)	1347 (36.6)	2276 (51.0)	3.8 (−6.8 to 19.1)	.52	4.9 (−5.2 to 22.6)	.49

^a^ Analysis of the Pregnancy Risk Assessment Monitoring System (PRAMS) data from 14 states and New York City (N = 11 432). Sample includes low-income women at 100% to 138% of the federal poverty level. Data were weighted using PRAMS sample weights. The model was adjusted for mother’s age, marital status, race and ethnicity, educational attainment, state-level rates of unemployment for women, and state and year fixed effects.

^b^ *P* values and confidence intervals were derived from wild bootstrap cluster SEs by state.

**Figure 2.**
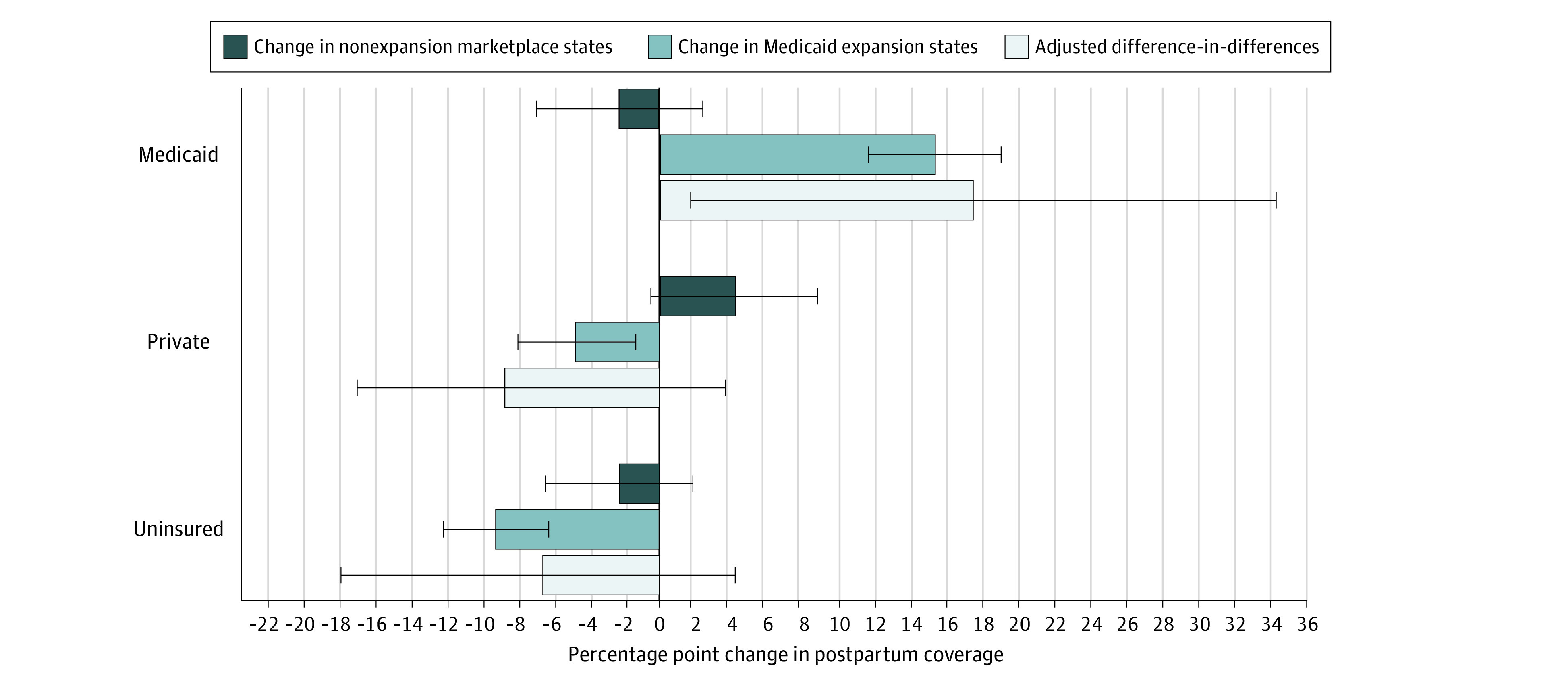
Changes in Postpartum Coverage Among Low-Income Women at 100% to 138% of the Federal Poverty Level, by Medicaid or Marketplace State Eligibility Analysis of Pregnancy Risk Assessment Monitoring System (PRAMS) data from 14 states and New York City (N = 11 432). Data were weighted using PRAMS sample weights. Error bars represent 95% CIs.

In the postpartum period, Medicaid relative to marketplace eligibility increased Medicaid coverage by 17.4 percentage points (95% CI, 1.7 to 34.3 percentage points) (Figure 2). We found no evidence of statistically significant changes in postpartum private coverage and uninsurance (Table 2).

Table 2 summarizes the difference-in-difference estimates for prenatal and postpartum care use. Medicaid relative to marketplace eligibility increased receipt of adequate prenatal care by 4.4 percentage points (95% CI, 0.1 to 11.0 percentage points) associated with a small increase in the number of prenatal care visits (0.4 additional prenatal care visits) (eTable 11 in the Supplement). We found no differences in early prenatal care, postpartum checkups, or contraceptive use.

In sensitivity analyses, including the transition year, excluding women aged 18 to 26 years, omitting states with up to at least 100% of the FPL pre-ACA Medicaid eligibility, and using a complete case analysis for missing data produced results largely consistent with the main models (eTables 5-8 in the Supplement). In addition, because our sample of women with low incomes would have qualified for pregnancy-related Medicaid coverage regardless of the ACA insurance expansions for their pregnancy duration and up to 60 days after childbirth, we excluded women within 2 months of childbirth as an additional sensitivity analysis and found results similar to those of the main models (eTable 9 in the Supplement).

## Discussion

In our analysis of women with low incomes (100%-138% FPL), we found that residence in Medicaid expansion states was associated with higher Medicaid coverage, lower private coverage, and lower preconception uninsurance. We also found that Medicaid eligibility was associated with increased use of adequate prenatal care, but found no evidence of differences in early prenatal care, postpartum visits, or postpartum contraceptive use.

Consistent with past research indicating lower uninsurance among individuals with low incomes eligible for Medicaid vs marketplace coverage, we found that Medicaid-eligible women had lower rates of preconception uninsurance relative to marketplace-eligible women.^4^ Women with incomes 100% to 138% of the FPL qualify for pregnancy Medicaid in all states and as a result, rates of uninsurance are very low at delivery and we identified no differences in uninsurance at childbirth between Medicaid- and marketplace-eligible states. High uninsurance rates before and after pregnancy suggest that women with low incomes face barriers to marketplace enrollment. This difficulty could be a result of restrictions on enrollment timing and/or the affordability of marketplace coverage. Research suggests low financial protection in marketplace plans, which could be contributing to lower enrollment among the marketplace-eligible population and is further supported by reports of marketplace eligibility among 1 in 5 uninsured adults.^18,19^ Medicaid eligibility allows women with low incomes to enroll at any time at no cost. However, women can only enroll in marketplace coverage during open enrollment or special enrollment periods (eg, birth and job loss, but not pregnancy), thus leaving many uninsured women unable to enroll in marketplace coverage when they are planning pregnancy. Furthermore, premium contributions for marketplace coverage for families at 100% to 138% of the FPL are up to 2% of income for the second-lowest-cost silver plan. This level would translate to an expected contribution of up to $626.44 with a maximum monthly premium payment of $39 to $52 for a family of 4.^20^ Under the American Rescue Plan, people with incomes under 150% of the FPL will now qualify for zero-dollar premiums for silver plans through the marketplace.^21^ Improved affordability of marketplace plans under the American Rescue Plan has the potential to address the higher preconception and postpartum uninsurance that we observed among marketplace-eligible women compared to Medicaid-eligible women.

Previous research has found that preconception coverage affects prenatal care use, including early prenatal care initiation and receipt of adequate prenatal care.^22,23^ In addition, coverage after pregnancy can affect health care use in the postpartum period. Past research found increased postpartum Medicaid coverage under the ACA Medicaid expansions were associated with increased postpartum outpatient care use as well as increased use of effective postpartum contraception.^6,24^ Although women in our study would have qualified for pregnancy-related Medicaid coverage regardless of the ACA insurance expansions during their pregnancies and up to 60 days after childbirth, the research indicating effects of income-based Medicaid expansions on prenatal and postpartum care use suggest that continuous Medicaid eligibility can increase maternal health care use beyond care provided under pregnancy-related Medicaid eligibility.^6,22,23,24^ Furthermore, research found increases in Medicaid coverage at delivery were associated with the ACA Medicaid expansions, suggesting effects of continuous Medicaid eligibility on pregnancy coverage despite the existing availability of pregnancy-related Medicaid eligibility.^10^ As a result, we may expect to see differences in prenatal and postpartum care use and maternal coverage in our study despite no changes to pregnancy-related Medicaid coverage under the ACA.

Although we found higher rates of adequate prenatal care among women eligible for Medicaid relative to marketplace coverage, it is unclear whether the small magnitude of the increase in the number of prenatal visits (0.4 additional visits) is clinically meaningful (eTable 11 in the Supplement). Research has demonstrated that prenatal care services can improve some maternal-child health outcomes.^25^ However, the importance of the timing and frequency of prenatal care, in particular, adhering to national guidelines that recommend 12 to14 in-person prenatal visits (on which the adequacy of prenatal care measure is based), is unclear.^25^

Because this study examined insurance eligibility and not enrollment, we are unable to distinguish whether the null differences in early prenatal care and postpartum care reflect that Medicaid and marketplace coverage provide similar access and quality of care, or alternative explanations such as lower access or quality of care among Medicaid-eligible women being offset by higher levels of uninsurance among marketplace-eligible women. Furthermore, we examined differences in preventive services (prenatal care, postpartum visits, and contraception) not subject to deductibles under marketplace plans. There are limited variables in PRAMS to examine nonpreventive health services used prior to or after pregnancy; thus, we cannot rule out meaningful differences in the use of services such as specialty care, ultrasonography, laboratory tests, or prescription medicines.

Similarly, the outcomes we examine do not capture all maternal health outcomes that could be affected by insurance eligibility, including maternal morbidity and mortality.^2,3^ We also have insufficient sample size to examine subgroup differences by race and ethnicity or other characteristics, which could mask differences within specific groups.

As of 2019, Medicaid financed 42.1% of births in the US.^26^ Thus, even small disparities in health care access and outcomes for Medicaid-eligible women would have far-reaching implications for maternal and infant health. Disparities for Medicaid-eligible women may also affect maternal and child health disparities by income and race and ethnicity, because Medicaid disproportionately serves low-income and racial and ethnic minority populations.

Evidence on the tradeoffs of expanding public vs private coverage for promoting maternal health is particularly policy relevant given the possibility of a public option under the Biden presidency and recent action to expand subsidies to improve the affordability of marketplace coverage.^27^ This research can also inform state decisions to adopt new American Rescue Plan policy options, namely the newly incentivized ACA Medicaid expansion, and the pregnancy Medicaid extension through 1-year post partum.^28^ Our results suggest that policy decisions to expand Medicaid relative to marketplace eligibility will contribute to improved insurance coverage for women with low incomes before and after pregnancy, although this differential could be attenuated given expanded marketplace subsidies.^29^

### Limitations

This study has several limitations. First, this identification strategy relies on the accuracy of the PRAMS survey data. Research has found generally high levels of agreement when comparing PRAMS with other data, but lower levels of agreement for the postpartum visits measure.^30,31,32,33^ However, assuming any bias in the measures is not differential between marketplace-eligible and Medicaid-eligible women, this lower level should not bias our difference-in-difference estimates; however, the overall rates of postpartum visits should be interpreted with caution. The potential for recall error for annual income in this survey, asked regarding the 12-month period before childbirth, could affect proper identification of the sample. Recall error could potentially lead to including some respondents who are outside of the income range in the sample, which could dampen the results. However, these data have been used in studies that rely on income measure to determine insurance eligibility.^7^ Furthermore, when using the upper-bound and lower-bound income estimates for each category instead of the midpoint, we observed similar coverage patterns as main models (eTable 4 in the Supplement). Second, owing to inconsistency in PRAMS reporting, analyses were limited to 14 states and New York City, and therefore may not be generalizable to women residing in dissimilar states, including southeastern states, because our sample included no states from this region. Third, these findings are only generalizable to the tradeoffs of public vs private eligibility for the low-income population between 100% and 138% of the FPL. However, this low-income population is a highly policy relevant population because this is the income range for which private and public eligibility continues to differ substantially across states. Fourth, the questions for private coverage differ across PRAMS phases and years, and questions do not distinguish between actuarial value of private plans (eTable 12 in the Supplement). Furthermore, postpartum coverage measures are only available in PRAMS beginning in 2012 and therefore have a shorter preperiod to examine the plausibility of the parallel-trends assumption. Fifth, the study was underpowered to examine infant outcomes, which may be affected by differences in preconception coverage and access to prenatal care.

## Conclusions

Eligibility for Medicaid compared with marketplace coverage resulted in decreased preconception uninsurance, a small increase in adequate prenatal care, and no difference in early prenatal care or postpartum care. The considerably lower rates of preconception uninsurance among Medicaid-eligible women underscores the importance of reducing financial barriers to coverage for the low-income population. Medicaid expansion or additional subsidies for marketplace coverage—such as those in the American Rescue Plan—may be a useful mechanism to improve coverage before and after pregnancy for women with low incomes.

## Notes

Supplement. **eTable 1.** List of States Included and Insurance Eligibility Category for Income 100%-138% FPL During Study Period **eTable 2.** Difference-in-Difference Model and Assumptions **eFigure 1.** Unadjusted Trends in Preconception and Postpartum Medicaid and Private Coverage Among Low-Income Women 100%-138% FPL, by Eligibility for Medicaid or Marketplace Insurance **eTable 3.** Prepolicy Placebo Tests **eFigure 2.** Plots of Placebo Test Coefficients **eTable 4.** Estimates of the Effects of Medicaid Versus Marketplace Eligibility on Maternal Coverage and Access to Prenatal and Postpartum Care, Using Lower-Bound and Upper-Bound Income Specifications **eTable 5.** Estimates of the Effects of Medicaid Versus Marketplace Eligibility on Maternal Coverage and Access to Prenatal and Postpartum Care, Including Transition Year **eTable 6.** Estimates of the Effects of Medicaid versus Marketplace Eligibility on Maternal Coverage and Access to Prenatal and Postpartum Care, Excluding Women Aged 18-26 Years **eTable 7.** Estimates of the Effects of Medicaid Versus Marketplace Eligibility on Maternal Coverage and Access to Prenatal and Postpartum Care, Omitting States with Generous Pre-ACA Medicaid Eligibility **eTable 8.** Estimates of the Effects of Medicaid Versus Marketplace Eligibility on Maternal Coverage and Access to Prenatal and Postpartum Care, Complete Case Analysis **eTable 9.** Estimates of the Effects of Medicaid Versus Marketplace Eligibility on Maternal Coverage and Access to Prenatal and Postpartum Care, Excluding Women with PRAMS Follow-up within 2 Months **eTable 10.** Estimates of the Effects of Medicaid Versus Marketplace Eligibility on Coverage at Delivery **eTable 11.** Estimates of the Effects of Medicaid Versus Marketplace Eligibility on Number of Prenatal Care Visits **eTable 12.** PRAMS Private Coverage Variables Over Time
