# Wording Error in Title

**DOI:** 10.1001/jamanetworkopen.2021.46242

**Published:** 2022-01-06

In the Original Investigation titled “Association of Medicaid vs Marketplace Eligibility on Maternal Coverage and Access With Prenatal and Postpartum Care,”^1^ published December 6, 2021, there was a wording error in the title. The title should have been “Association of Medicaid vs Marketplace Eligibility With Maternal Coverage and Access to Prenatal and Postpartum Care.” This article and its supplement have been corrected.^1^
