# Supplementary material for: Association of Medicaid vs Marketplace Eligibility on Maternal Coverage and Access With Prenatal and Postpartum Care
Source: JAMA Netw Open. 2021 Dec 6;4(12):e2137383. doi: 10.1001/jamanetworkopen.2021.37383 (PMC8649838; doi:10.1001/jamanetworkopen.2021.37383)
Supplement: Supplement. — eTable 1. List of States Included and Insurance Eligibility Category for Income 100%-138% FPL During Study Period eTable 2. Difference-in-Difference Model and Assumptions eFigure 1. Unadjusted Trends in Preconception and Postpartum Medicaid and Private Coverage Among Low-Income Women 100%-138% FPL, by Eligibility for Medicaid or Marketplace Insurance eTable 3. Prepolicy Placebo Tests eFigure 2. Plots of Placebo Test Coefficients eTable 4. Estimates of the Effects of Medicaid Versus Marketplace Eligibility on Maternal Coverage and Access to Prenatal and Postpartum Care, Using Lower-Bound and Upper-Bound Income Specifications eTable 5. Estimates of the Effects of Medicaid Versus Marketplace Eligibility on Maternal Coverage and Access to Prenatal and Postpartum Care, Including Transition Year eTable 6. Estimates of the Effects of Medicaid versus Marketplace Eligibility on Maternal Coverage and Access to Prenatal and Postpartum Care, Excluding Women Aged 18-26 Years eTable 7. Estimates of the Effects of Medicaid Versus Marketplace Eligibility on Maternal Coverage and Access to Prenatal and Postpartum Care, Omitting States with Generous Pre-ACA Medicaid Eligibility eTable 8. Estimates of the Effects of Medicaid Versus Marketplace Eligibility on Maternal Coverage and Access to Prenatal and Postpartum Care, Complete Case Analysis eTable 9. Estimates of the Effects of Medicaid Versus Marketplace Eligibility on Maternal Coverage and Access to Prenatal and Postpartum Care, Excluding Women with PRAMS Follow-up within 2 Months eTable 10. Estimates of the Effects of Medicaid Versus Marketplace Eligibility on Coverage at Delivery eTable 11. Estimates of the Effects of Medicaid Versus Marketplace Eligibility on Number of Prenatal Care Visits eTable 12. PRAMS Private Coverage Variables Over Time [file jamanetwopen-e2137383-s001.pdf]

Supplementary Online Content

Eliason EL, Daw JR, Allen HL. Association of Medicaid vs marketplace eligibility on maternal coverage and access with prenatal and postpartum care. *JAMA Netw Open*. 2021;4(12):e2137383. doi:10.1001/jamanetworkopen.2021.37383

eTable 1. List of States Included and Insurance Eligibility Category for Income 100%-138% FPL During Study Period
eTable 2. Difference-in-Difference Model and Assumptions
eFigure 1. Unadjusted Trends in Preconception and Postpartum Medicaid and Private Coverage Among Low-Income Women 100%-138% FPL, by Eligibility for Medicaid or Marketplace Insurance
eTable 3. Prepolicy Placebo Tests
eFigure 2. Plots of Placebo Test Coefficients
eTable 4. Estimates of the Effects of Medicaid Versus Marketplace Eligibility on Maternal Coverage and Access to Prenatal and Postpartum Care, Using Lower-Bound and Upper-Bound Income Specifications
eTable 5. Estimates of the Effects of Medicaid Versus Marketplace Eligibility on Maternal Coverage and Access to Prenatal and Postpartum Care, Including Transition Year
eTable 6. Estimates of the Effects of Medicaid Versus Marketplace Eligibility on Maternal Coverage and Access to Prenatal and Postpartum Care, Excluding Women Aged 18-26 Years
eTable 7. Estimates of the Effects of Medicaid Versus Marketplace Eligibility on Maternal Coverage and Access to Prenatal and Postpartum Care, Omitting States with Generous Pre-ACA Medicaid Eligibility
eTable 8. Estimates of the Effects of Medicaid Versus Marketplace Eligibility on Maternal Coverage and Access to Prenatal and Postpartum Care, Complete Case Analysis
eTable 9. Estimates of the Effects of Medicaid Versus Marketplace Eligibility on Maternal Coverage and Access to Prenatal and Postpartum Care, Excluding Women with PRAMS Follow-up within 2 Months
eTable 10. Estimates of the Effects of Medicaid Versus Marketplace Eligibility on Coverage at Delivery
eTable 11. Estimates of the Effects of Medicaid Versus Marketplace Eligibility on Number of Prenatal Care Visits
eTable 12. PRAMS Private Coverage Variables Over Time

This supplementary material has been provided by the authors to give readers additional information about their work.

© 2021 Eliason EL et al. *JAMA Network Open.*

eTable1. List of States Included and Insurance Eligibility Category for Income 100%-138% FPL during Study Period

Medicaid Expansion States	Marketplace States
Colorado, Delaware, Massachusetts, New Jersey, New Mexico, New York City, Pennsylvania, Vermont, Washington, West Virginia	Maine, Missouri, Utah, Wisconsin, Wyoming

© 2021 Eliason EL et al. *JAMA Network Open.*

eTable2. Difference-in-Difference Model and Assumptions

Our model is specified below:

$y_{it} = \beta 0 + \beta 1(Post*Expansion)_{it} + (YearFE)_t + (StateFE)_i + \gamma` x_{it} + \varepsilon_{it}$

The β1 coefficient estimates changes in outcomes among women gaining Medicaid eligibility relative to Marketplace eligibility after policy implementation. Y_{it} represents the dependent variable. $\gamma`\mathbf{x}_{it}$ is a vector of control variables, including the state unemployment rate for women from the Bureau of Labor Statistics, mother's age, marital status, race, Hispanic ethnicity, and high school educational attainment.

The table below presents an analysis of the difference-in-differences assumption that the trends prior to policy implementation are similar for mothers gaining Medicaid and Marketplace eligibility. This pre-policy trend comparison follows the approach of other health insurance studies that tests pre-trends for difference-in-differences models. Analysis is limited to prior to 2014 to compare parallel trends in the pre-period, and each outcome is modeled as an interaction of a linear time trend and treatment group status to assess significant differences in trends prior to the policy implementation. The β2 coefficient, presented in the table, indicates any significant difference in pre-policy trends between mothers gaining Medicaid and Marketplace eligibility, as specified:

$y_{it} = \beta 0 + \beta 1 LinearTimeTrend_t + \beta 2 TreatmentStatus_i * LinearTimeTrend_t + \mathbf{(StateFE)_i} + \gamma`\mathbf{x}_{it} + + \varepsilon_{it}$

Dependent Variable:	Trend Difference	*P*-Value
Preconception Medicaid Coverage	-0.006	0.788
Preconception Private Insurance	-0.015	0.554
No Preconception Insurance	0.015	0.550
Postpartum Medicaid Coverage	0.061	0.210
Postpartum Private Insurance	-0.005	0.910
No Postpartum Insurance	-0.052	0.215
Early Prenatal Care	0.005	0.834
Adequate Prenatal Care	0.027	0.289
Postpartum Check-Up	-0.008	0.662
Effective Postpartum Contraception	-0.019	0.396

Notes: Authors' analysis of Pregnancy Risk Assessment Monitoring System data from fourteen states and New York City. Sample includes low-income women 100-138 percent of the federal poverty level. Data are weighted using PRAMS sample weights.

© 2021 Eliason EL et al. *JAMA Network Open.*

eFigure1. Unadjusted Trends in Preconception and Postpartum Medicaid and Private Coverage among Low-Income Women 100-138% FPL, by Eligibility for Medicaid or Marketplace Insurance

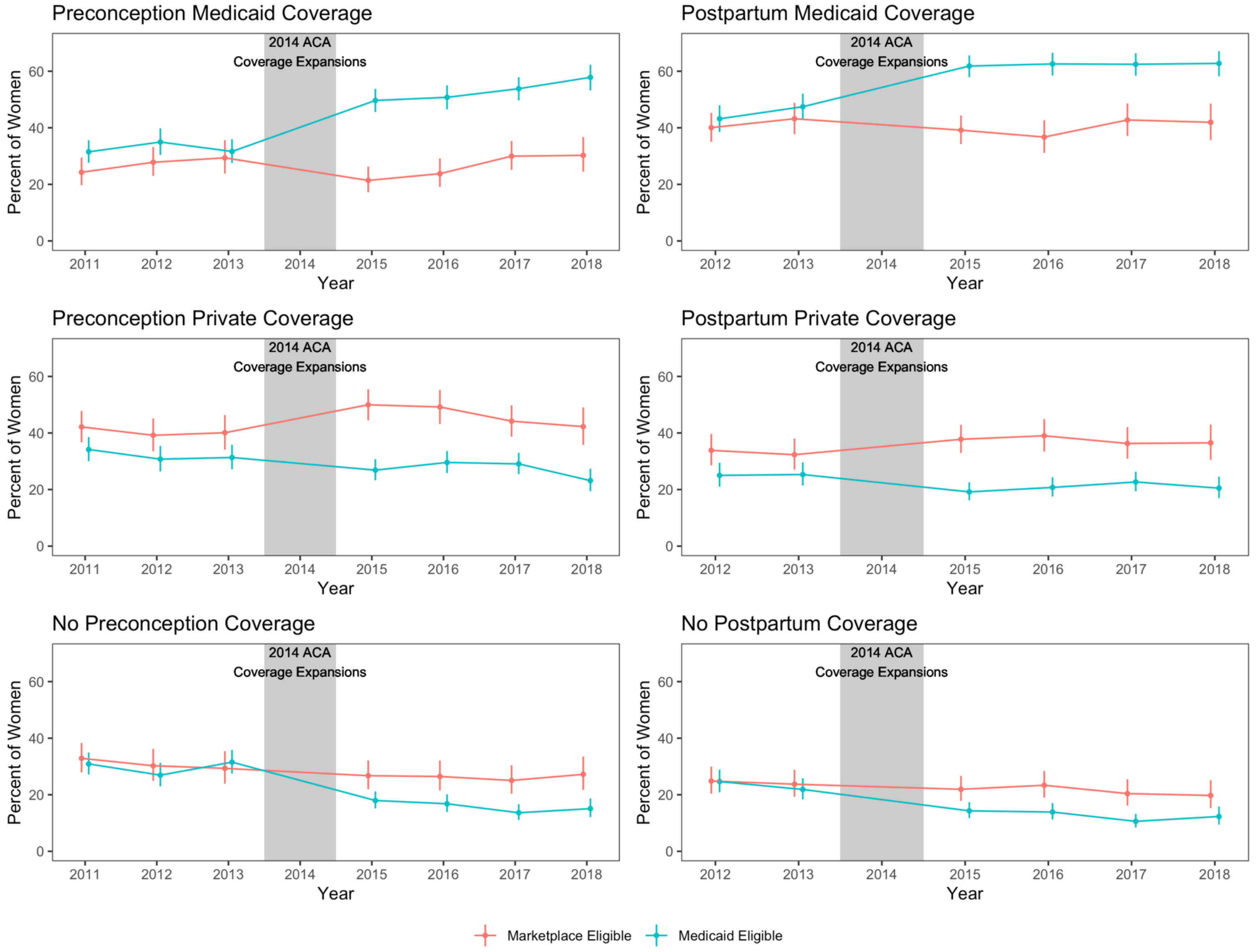

© 2021 Eliason EL et al. *JAMA Network Open.*

eTable3. Prepolicy Placebo Tests

Dependent Variable:		Coefficient	Lower bound 95% CI	Upper bound 95% CI	F-Statistic	*P-Value*
Preconception Coverage						
Medicaid	2011	1.08	-2.82	4.99	0.30	0.748
	2012	0.81	-6.54	8.17		
Private	2011	3.29	-6.55	13.13	0.40	0.677
	2012	2.89	-7.07	12.85		
None	2011	-2.83	-10.57	4.91	0.33	0.725
	2012	-2.82	-14.29	8.65		
Postpartum Coverage						
Medicaid	2012	-6.14	-15.73	3.45	1.57	0.210
Private	2012	0.55	-9.03	10.13	0.01	0.910
None	2012	5.25	-3.04	1.35	1.54	0.215
Access and Childbirth Outcomes						
Early Prenatal Care	2011	-1.10	-8.40	6.21	0.54	0.594
	2012	-3.41	-10.86	4.04		
Adequate Prenatal Care	2011	-5.93	-13.88	2.01	1.60	0.237
	2012	-0.50	-13.04	12.03		
Postpartum Check-Up	2011	1.04	-5.07	7.15	3.37	0.064
	2012	4.35	-3.06	11.18		
Effective Postpartum Contraception	2011	2.21	-25.42	29.85	0.96	0.409
	2012	-9.95	-25.57	5.67		

Notes: Authors' analysis of Pregnancy Risk Assessment Monitoring System data from fourteen states and New York City. Sample includes low-income women 100-138 percent of the federal poverty level. Placebo test coefficients represent the difference in the outcome in Medicaid states relative to Marketplace states in each year compared to the last year of the pre-policy period (2013). Data are weighted using PRAMS sample weights. Estimates expressed as percentage points. Estimates are adjusted for mother's age, marital status, race/ethnicity, educational attainment, state level rates of unemployment for women, and state and year fixed effects. F-statistics represent joint tests of the null hypothesis that all pre-policy interaction terms are equal to zero.

© 2021 Eliason EL et al. *JAMA Network Open.*

eFigure2. Plots of Placebo Test Coefficients

Estimates expressed as percentage points. Error bars represent 95% CIs. Placebo test coefficients represent the difference in the outcome in Medicaid states relative to Marketplace states in each year compared to the last year of the pre-policy period (2013). Data are weighted using PRAMS sample weights. Estimates are adjusted for mother's age, marital status, race/ethnicity, educational attainment, state level rates of unemployment for women, and state and year fixed effects. Authors' analysis of Pregnancy Risk Assessment Monitoring System data from fourteen states and New York City. Sample includes low-income women 100-138 percent of the federal poverty level.

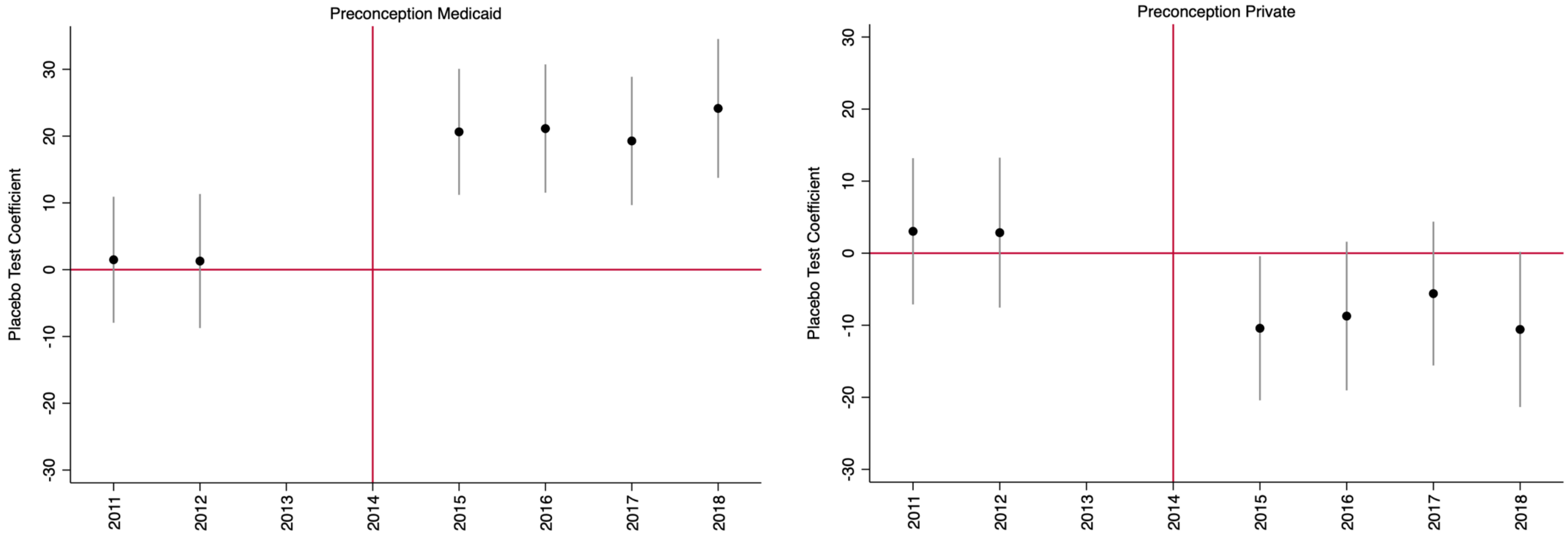

© 2021 Eliason EL et al. *JAMA Network Open.*

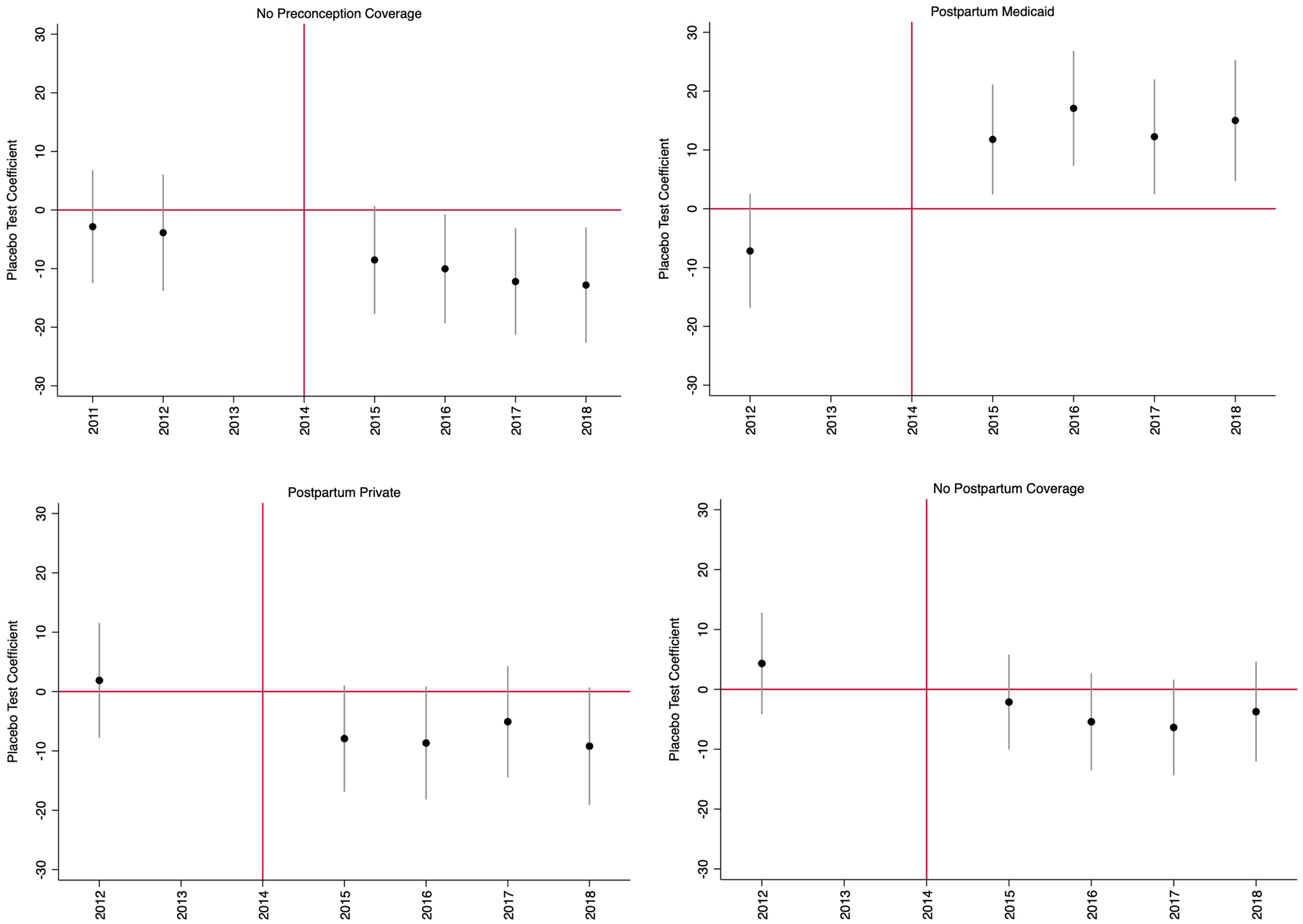

© 2021 Eliason EL et al. *JAMA Network Open.*

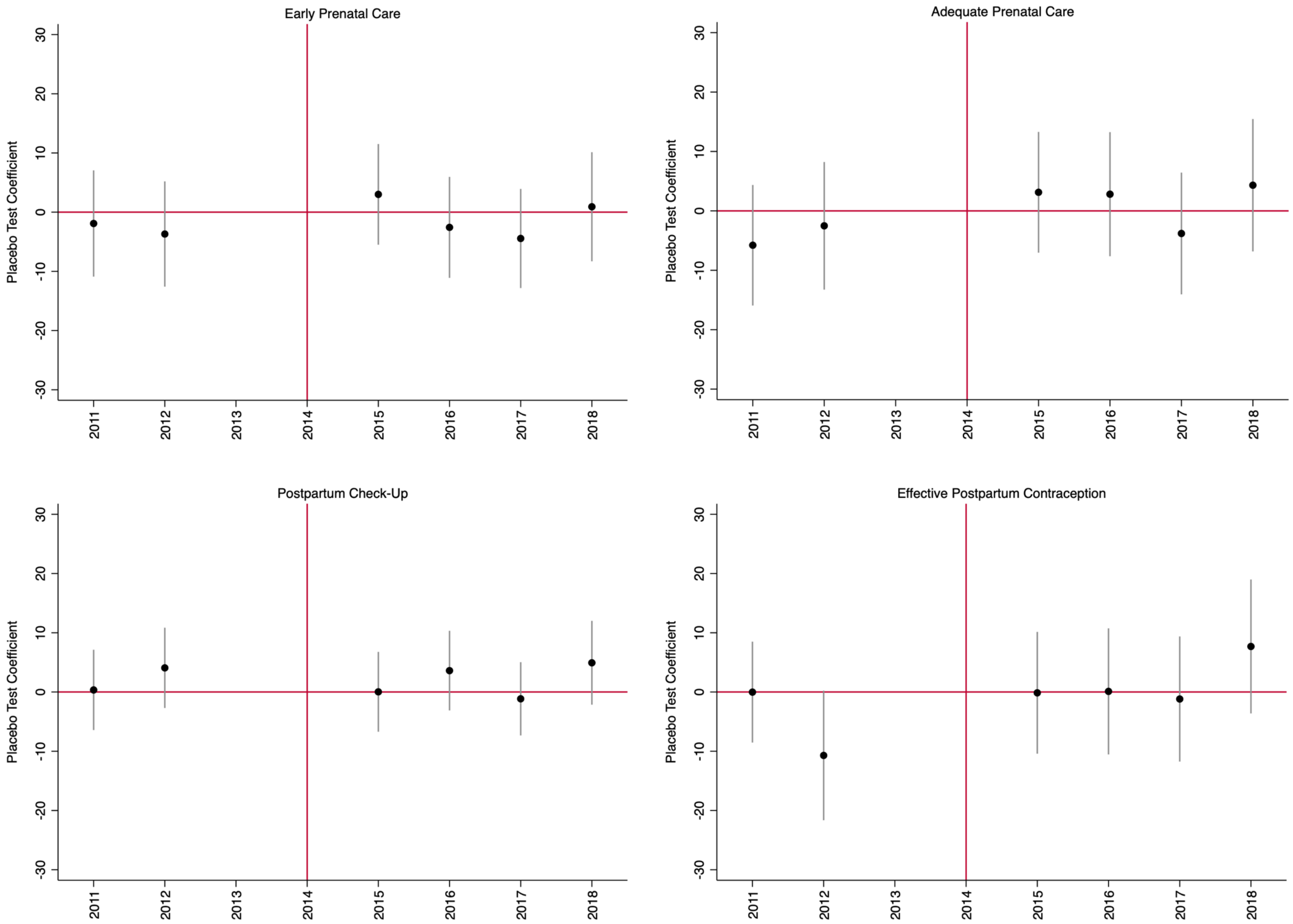

© 2021 Eliason EL et al. *JAMA Network Open.*

eTable4. Estimates of the Effects of Medicaid versus Marketplace Eligibility on Maternal Coverage and Access to Prenatal and Postpartum Care, Using Lower-Bound and Upper-Bound Income Specifications

	Adjusted Effects of Medicaid versus Marketplace Eligibility	
Dependent Variable:	Lower-Bound Income Specifications (N=10,588)	Upper-Bound Income Specifications (N=15,449)
Preconception Coverage		
Medicaid	15.3*	20.5**
	(8.9, 24.6)	(12.6, 31.1)
	**0.010**	**0.005**
Private	-5.0	-11.1**
	(-14.6, 1.5)	(-17.2, -5.7)
	0.307	**0.005**
None	-10.0*	-9.7*
	(-20.2, -2.1)	(-22.0, -0.8)
	**0.018**	**0.040**
Postpartum Coverage		
Medicaid	17.8**	15.8***
	(6.8, 29.5)	(3.4, 30.4)
	**0.002**	**0.000**
Private	-9.0	-6.6
	(-20.5, 1.2)	(-15.5, 2.0)
	0.101	0.237
None	-7.0†	-7.4†
	(-19.7, 0.8)	(-21.0, 0.5)
	0.062	0.063
Early Prenatal Care	-1.0	2.8
	(-3.6, 2.0)	(-1.6, 8.9)
	0.441	0.224
Adequate Prenatal Care	-1.7	3.3
	(-7.3, 5.2)	(-1.7, 9.1)
	0.447	0.113
Postpartum Check-Up	-1.9	2.5
	(-9.2, 3.13	(-1.3, 6.6)
	0.666	0.323
Effective Postpartum Contraception	5.0	2.8
	(-16.2, 44.4)	(-7.7, 20.8)
	0.780	0.654

Notes: Authors' analysis of Pregnancy Risk Assessment Monitoring System data from fourteen states and New York City. Sample includes low-income women 100-138 percent of the federal poverty level. Data are weighted using PRAMS sample weights. Estimates are presented as percentage points. Model is adjusted for marital status, race/ethnicity, educational attainment, state level rates of unemployment for women, and state and year fixed effects. P-values and confidence intervals are derived from wild bootstrap cluster standard errors by state. Boldface indicates p<0.05. †p<0.10, *p<0.05, **p<0.01, ***p<0.001

© 2021 Eliason EL et al. *JAMA Network Open.*

eTable5. Estimates of the Effects of Medicaid versus Marketplace Eligibility on Maternal Coverage and Access to Prenatal and Postpartum Care, Including Transition Year

	Effects of Medicaid versus Marketplace Eligibility	
Dependent Variable:	Unadjusted Difference-in-Difference	Unadjusted Difference-in-Difference
Preconception Coverage		
Medicaid	12.8†	12.8†
	(-0.1, 25.9)	(-1.4, 25.5)
	0.051	0.058
Private	-7.6**	-8.0*
	(-12.5, -1.9)	(-12.5, -0.3)
	**0.005**	**0.045**
None	-4.7	-4.4
	(-15.0, 2.3)	(-14.8, 3.6)
	0.194	0.273
Postpartum Coverage		
Medicaid	13.1**	12.0**
	(3.1, 34.0)	(2.0, 31.5)
	**0.008**	**0.007**
Private	-7.6	-7.7
	(-15.7, 3.8)	(-15.5, 3.6)
	0.127	0.150
None	-3.9	-2.9
	(-14.0, 4.7)	(-11.9, 5.3)
	0.351	0.477
Early Prenatal Care	1.2	0.7
	(-3.9, 5.4)	(-4.6, 5.1)
	0.613	0.763
Adequate Prenatal Care	5.7*	5.2*
	(0.2, 10.7)	(0.1, 10.1)
	**0.044**	**0.044**
Postpartum Check-Up	0.6	0.2
	(-1.8, 3.9)	(-2.0, 3.7)
	0.685	0.884
Effective Postpartum Contraception	4.0	4.0
	(-5.8, 15.8)	(-5.1, 15.9)
	0.393	0.380

Notes: Authors' analysis of Pregnancy Risk Assessment Monitoring System data from fourteen states and New York City. Sample includes low-income women 100-138 percent of the federal poverty level. Data are weighted using PRAMS sample weights. Estimates are presented as percentage points. Model is adjusted for marital status, race/ethnicity, educational attainment, state level rates of unemployment for women, and state and year fixed effects. P-values and confidence intervals are derived from wild bootstrap cluster standard errors by state. Boldface indicates p<0.05. †p<0.10, *p<0.05, **p<0.01, ***p<0.001

© 2021 Eliason EL et al. *JAMA Network Open.*

eTable6. Estimates of the Effects of Medicaid versus Marketplace Eligibility on Maternal Coverage and Access to Prenatal and Postpartum Care, Excluding Women Aged 18-26 Years

	Medicaid Non-expansion States (*Marketplace Eligible*)		Medicaid Expansion States (*Medicaid Eligible*)		Effects of Medicaid versus Marketplace Eligibility	
Dependent Variable:	Pre-policy (N=821)	Post-policy (N=1,214)	Pre-policy (N=1,875)	Post-policy (N=2,624)	Unadjusted Difference-in-Difference Estimate	Adjusted Difference-in-Difference Estimate
Preconception Coverage						
Medicaid	25.7	25.8	33.0	51.9	13.1**	20.1***
					(2.8, 25.7)	(9.8, 33.1)
					**0.008**	**0.000**
Private	43.6	46.1	32.7	26.5	-8.5**	-10.0*
					(-13.6, -4.2)	(-13.4, -4.4)
					**0.003**	**0.010**
None	29.2	27.6	28.2	17.2	-4.6	-9.7†
					(-16.9, 4.9)	(-23.5, 1.9)
					0.372	0.059
Postpartum Coverage						
Medicaid	36.9	37.9	44.7	59.0	12.5***	14.3*
					(5.0, 25.2)	(2.8, 32.5)
					**0.000**	**0.010**
Private	36.5	39.5	24.7	21.9	-6.2	-7.1
					(-15.5, 4.9)	(-18.5, 4.3)
					0.214	0.267
None	26.3	22.0	21.6	14.4	-3.2	-2.9
					(-8.4, 5.5)	(-13.1, 9.3)
					0.217	0.446
Access and Childbirth Outcomes						
Early Prenatal Care	81.7	83.6	80.1	83.8	-0.1	1.7
					(-5.6, 7.8)	(-2.3, 8.9)
					0.968	0.465

© 2021 Eliason EL et al. *JAMA Network Open.*

Kessner Prenatal Care Index	78.3	77.0	67.3	70.7	5.7†	4.3†
					(-0.8, 13.2)	(-0.1, 9.4)
					0.080	0.069
Postpartum Check-Up	91.5	88.5	90.3	89.1	1.4	0.9
					(-2.4, 4.5)	(-4.5, 4.8)
					0.447	0.723
Effective Postpartum Contraception	38.6	51.3	31.4	48.4	2.2	5.2
					(-9.9, 24.8)	(-11.4, 30.4)
					0.780	0.594

Notes: Authors' analysis of Pregnancy Risk Assessment Monitoring System data from fourteen states and New York City. Sample includes low-income women 100-138 percent of the federal poverty level. Women 18-26 years old are identified using birth year and year of survey, as maternal age is measured through categories that do not align with excluding women under the age of 26, and may therefore not perfect capture women in this age group. Data are weighted using PRAMS sample weights. Estimates are presented as percentage points. Model is adjusted for marital status, race/ethnicity, educational attainment, state level rates of unemployment for women, and state and year fixed effects. P-values and confidence intervals are derived from wild bootstrap cluster standard errors by state. Boldface indicates p<0.05. †p<0.10, *p<0.05, **p<0.01, ***p<0.001

© 2021 Eliason EL et al. *JAMA Network Open.*

eTable7. Estimates of the Effects of Medicaid versus Marketplace Eligibility on Maternal Coverage and Access to Prenatal and Postpartum Care, Omitting States with Generous Pre-ACA Medicaid Eligibility

	Medicaid Non-expansion States (*Marketplace Eligible*)		Medicaid Expansion States (*Medicaid Eligible*)		Effects of Medicaid versus Marketplace Eligibility	
Dependent Variable:	Pre-policy (N=1,645)	Post-policy (N=2,201)	Pre-policy (N=2,092)	Post-policy (N=2,560)	Unadjusted Difference-in-Difference	Adjusted Difference-in-Difference
Preconception Coverage						
Medicaid	27.1	25.4	26.4	48.4	23.1**	23.4**
					(13.8, 32.7)	(14.4, 32.1)
					**0.008**	**0.006**
Private	40.5	46.3	35.1	30.4	-10.2**	-10.5**
					(-14.8, -4.7)	(-13.9, -6.4)
					**0.004**	**0.001**
None	30.8	27.3	35.2	18.2	-12.8*	-12.7*
					(-25.9, -0.7)	(-23.4, -1.5)
					**0.035**	**0.037**
Postpartum Coverage						
Medicaid	41.7	39.5	41.4	58.0	19.0*	19.4*
					(1.8, 41.6)	(0.8, 36.8)
					**0.026**	**0.040**
Private	33.1	37.3	28.1	22.7	-9.0	-9.3
					(-18.9, 5.6)	(-18.4, 4.25)
					0.237	0.228
None	24.3	22.0	27.7	15.9	-10.3†	-10.3*
					(-22.5, 1.91)	(-21.1, -0.1)
					0.092	**0.042**

© 2021 Eliason EL et al. *JAMA Network Open.*

Early Prenatal Care	80.8	83.4	79.2	83.6	1.6	1.2
					(-4.3, 6.4)	(-5.2, 7.9)
					0.510	0.685
Kessner Prenatal Care Index	75.4	74.2	63.3	67.9	5.6*	4.7*
					(1.1, 12.1)	(0.3, 10.8)
					**0.022**	**0.042**
Postpartum Check-Up	90.4	86.9	89.1	88.0	1.3	0.9
					(-2.8, 5.4)	(-2.9, 5.2)
					0.560	0.741
Effective Postpartum Contraception	43.5	54.2	36.5	53.3	6.0	6.6
					(-5.5, 21.4)	(-5.1, 23.9)
					0.324	0.320

Notes: Authors' analysis of Pregnancy Risk Assessment Monitoring System data from eleven states. Sample includes low-income women 100-138 percent of the federal poverty level. Data are weighted using PRAMS sample weights. Estimates are presented as percentage points. Model is adjusted for marital status, race/ethnicity, educational attainment, state level rates of unemployment for women, and state and year fixed effects. P-values and confidence intervals are derived from wild bootstrap cluster standard errors by state. Boldface indicates p<0.05. †p<0.10, *p<0.05, **p<0.01, ***p<0.001

© 2021 Eliason EL et al. *JAMA Network Open.*

eTable8. Estimates of the Effects of Medicaid versus Marketplace Eligibility on Maternal Coverage and Access to Prenatal and Postpartum Care , Complete Case Analysis

	Medicaid Non-expansion States (*Marketplace Eligible*)		Medicaid Expansion States (*Medicaid Eligible*)		Effects of Medicaid versus Marketplace Eligibility	
Dependent Variable:	Pre-policy (N=1,594)	Post-policy (N=2,138)	Pre-policy (N=2,962)	Post-policy (N=3,797)	Unadjusted Difference-in-Difference Estimate	Adjusted Difference-in-Difference Estimate
Preconception Coverage						
Medicaid	27.0	25.7	32.9	49.7	19.6**	19.9**
					(11.9, 28.8)	(12.7, 28.6)
					**0.004**	**0.003**
Private	40.8	45.8	32.2	27.5	-9.4**	-10.0**
					(-12.6, -5.5)	(-12.1, -7.1)
					**0.002**	**0.002**
None	30.7	27.6	30.0	18.9	-9.2*	-9.1*
					(-21.5, -0.1)	(-20.4, -0.3)
					**0.047**	**0.046**
Postpartum Coverage						
Medicaid	41.7	40.0	46.2	61.6	17.2*	17.0*
					(2.6, 36.0)	(1.5, 33.2)
					**0.011**	**0.017**
Private	33.1	36.5	25.6	21.1	-7.4	-7.7
					(-15.9, 5.5)	(-15.8, 4.5)
					0.280	0.270
None	24.3	22.2	22.7	13.3	-7.5	-7.1
					(-18.5, 4.9)	(-17.4, 3.5)
					0.202	0.149

© 2021 Eliason EL et al. *JAMA Network Open.*

Early Prenatal Care	80.8	83.6	80.1	83.8	1.0	0.7
					(-3.9, 5.7)	(-4.4, 7.5)
					0.667	0.780
Kessner Prenatal Care Index	75.4	74.0	65.4	70.1	6.0*	5.1*
					(0.9, 12.4)	(0.9, 11.0)
					**0.033**	**0.032**
Postpartum Check-Up	90.7	87.1	89.7	87.8	0.7	0.4
					(-3.1, 4.1)	(-3.2, 3.9)
					0.693	0.808
Effective Postpartum Contraception	43.7	54.4	36.3	51.1	4.1	5.2
					(-7.9, 19.6)	(-5.7, 22.7)
					0.481	0.421

Notes: Authors' analysis of Pregnancy Risk Assessment Monitoring System data from fourteen states and New York City. Sample includes low-income women 100-138 percent of the federal poverty level. Data are weighted using PRAMS sample weights. Estimates are presented as percentage points. Model is adjusted for marital status, race/ethnicity, educational attainment, state level rates of unemployment for women, and state and year fixed effects. P-values and confidence intervals are derived from wild bootstrap cluster standard errors by state. Boldface indicates $p<0.05$. †$p<0.10$, *$p<0.05$, **$p<0.01$, ***$p<0.001$

© 2021 Eliason EL et al. *JAMA Network Open.*

eTable9. Estimates of the Effects of Medicaid versus Marketplace Eligibility on Maternal Coverage and Access to Prenatal and Postpartum Care, Excluding Women with PRAMS Follow-Up within 2 Months

	Medicaid Non-expansion States (*Marketplace Eligible*)		Medicaid Expansion States (*Medicaid Eligible*)		Effects of Medicaid versus Marketplace Eligibility	
Dependent Variable:	Pre-policy (N=1,547)	Post-policy (N=2,081)	Pre-policy (N=3,319)	Post-policy (N=4,143)	Unadjusted Difference-in-Difference	Adjusted Difference-in-Difference
Preconception Coverage						
Medicaid	27.3	26.9	33.4	53.1	19.1**	19.3**
					(11.0, 30.0)	(11.6, 30.3)
					**0.003**	**0.003**
Private	41.0	45.3	32.3	27.1	-8.7**	-9.1*
					(-13.7, -1.2)	(-12.8, -3.3)
					**0.006**	**0.010**
None	30.3	26.7	29.2	15.9	-9.3*	-9.1*
					(-20.4, -0.7)	(-20.0, -1.6)
					**0.039**	**0.031**
Postpartum Coverage						
Medicaid	41.5	40.0	46.4	62.2	16.5*	16.5†
					(0.0, 37.9)	(-1.5, 35.3)
					**0.050**	0.094
Private	33.1	37.2	25.6	20.9	-7.6	-7.9
					(-19.2, 8.3)	(-18.6, 7.2)
					0.275	0.257
None	24.4	21.7	22.2	12.8	-6.5	-6.2
					(-17.4, 5.4)	(-17.5, 4.7)
					0.240	0.196

© 2021 Eliason EL et al. *JAMA Network Open.*

Early Prenatal Care	80.8	82.4	80.1	84.2	2.1	1.7
					(-3.7, 6.3)	(-4.4, 8.2)
					0.377	0.538
Kessner Prenatal Care Index	75.8	73.5	65.5	70.2	6.6*	5.6*
					(1.3, 12.2)	(0.7, 10.7)
					**0.021**	**0.038**
Postpartum Check-Up	90.0	87.1	89.3	87.6	0.9	0.6
					(-3.0, 5.2)	(-3.0, 4.8)
					0.702	0.795
Effective Postpartum Contraception	44.3	53.7	37.2	50.7	4.3	5.5
					(-5.3, 18.7)	(-3.4, 22.7)
					0.482	0.436

Notes: Authors' analysis of Pregnancy Risk Assessment Monitoring System data from fourteen states and New York City. Sample includes low-income women 100-138 percent of the federal poverty level. Data are weighted using PRAMS sample weights. Estimates are presented as percentage points. Model is adjusted for marital status, race/ethnicity, educational attainment, state level rates of unemployment for women, and state and year fixed effects. P-values and confidence intervals are derived from wild bootstrap cluster standard errors by state. Boldface indicates p<0.05. †p<0.10, *p<0.05, **p<0.01, ***p<0.001

© 2021 Eliason EL et al. *JAMA Network Open.*

eTable10. Estimates of the Effects of Medicaid versus Marketplace Eligibility on Coverage at Delivery

	Medicaid Non-expansion States (*Marketplace Eligible*)		Medicaid Expansion States (*Medicaid Eligible*)		Effects of Medicaid versus Marketplace Eligibility	
Dependent Variable:	Pre-policy (N=1,645)	Post-policy (N=2,201)	Pre-policy (N=3,389)	Post-policy (N=4,197)	Unadjusted Difference-in-Difference Estimate	Adjusted Difference-in-Difference Estimate
Coverage at Delivery						
Medicaid	56.8	55.3	66.5	70.8	8.4*	9.2*
					(1.1, 14.3)	(2.1, 15.2)
					**0.032**	**0.031**
Private	38.7	40.0	28.5	24.9	-6.1†	-7.2*
					(-11.3, 0.8)	(-12.4, -0.2)
					0.093	**0.044**
None (Self-Pay)	4.1	4.4	2.4	3.5	-0.5	-0.3
					(-2.7, 2.1)	(-3.0, 2.2)
					0.657	0.813

Notes: Authors' analysis of Pregnancy Risk Assessment Monitoring System data from fourteen states and New York City. Sample includes low-income women 100-138 percent of the federal poverty level. Data are weighted using PRAMS sample weights. Model is adjusted for mother's age, marital status, educational attainment, state level rates of unemployment for women, and state and year fixed effects. P-values and confidence intervals are derived from wild bootstrap cluster standard errors by state. Boldface indicates p<0.05. †p<0.10, *p<0.05, **p<0.01, ***p<0.001

© 2021 Eliason EL et al. *JAMA Network Open.*

eTable11. Estimates of the Effects of Medicaid versus Marketplace Eligibility on Number of Prenatal Care Visits

	Medicaid Non-expansion States (*Marketplace Eligible*)		Medicaid Expansion States (*Medicaid Eligible*)		Effects of Medicaid versus Marketplace Eligibility	
Dependent Variable:	Pre-policy (N=1,645)	Post-policy (N=2,201)	Pre-policy (N=3,389)	Post-policy (N=4,197)	Unadjusted Difference-in-Difference Estimate	Adjusted Difference-in-Difference Estimate
Number of Prenatal Care Visits	9.4	9.3	8.4	8.7	0.5*	0.4*
					(0.001, 1.0)	(0.05, 1.0)
					**0.050**	**0.030**

Notes: Authors' analysis of Pregnancy Risk Assessment Monitoring System data from fourteen states and New York City. Sample includes low-income women 100-138 percent of the federal poverty level. Number of prenatal care visits are estimates from grouped number of prenatal visits (<= 8; 9-11; 12+). The lowest number is assigned for each category (1,9,12). Data are weighted using PRAMS sample weights. Model is adjusted for mother's age, marital status, educational attainment, state level rates of unemployment for women, and state and year fixed effects. P-values and confidence intervals are derived from wild bootstrap cluster standard errors by state. Boldface indicates p<0.05. †p<0.10, *p<0.05, **p<0.01, ***p<0.001

© 2021 Eliason EL et al. *JAMA Network Open.*

eTable12. PRAMS Private Coverage Variables Over Time

PRAMS Private Coverage Question	PRAMS Survey Year
health insurance that you or someone else paid for (not from a job)	2011
private health insurance purchased directly from an insurance company	2012-2015
private health insurance from the Health Insurance Marketplace or HealthCare.gov	2016-2018

Notes: PRAMS survey responses indicating non-group private insurance changed over the study period. As a result, our ability to capture specific increases in Marketplace coverage are limited by the change in this measure 2011-2018. Examining rates for this non-group private insurance separately from the broader private insurance category resulted in lower reports of non-group private insurance compared to other estimates for this population, such as those presented in Blavin F, Karpman M, Kenney GM, Sommers BD. Medicaid versus marketplace coverage for near-poor adults: Effects on out-of-pocket spending and coverage. *Health Aff (Millwood)*. 2018;37(2):299-307. doi:10.1377/hlthaff.2017.1166. For these reasons, we use a measure of private insurance in our analyses that encompasses all PRAMS private coverage variables.

© 2021 Eliason EL et al. *JAMA Network Open.*
